# Education Research: Perspectives and Experiences of Clinical Neurology Faculty Regarding the End-of-Rotation Assessment: A Qualitative Study

**DOI:** 10.1212/NE9.0000000000200104

**Published:** 2023-11-29

**Authors:** Marie Carl Eugene, Jose Montes-Rivera, Bobbie Ann Adair White

**Affiliations:** From the Neurology Department (M.C.E., J.M.-R.), UConn Health/UConn School of Medicine, Farmington, CT; and MGH Institute of Health Professions (B.A.A.W.), Boston, MA.

## Abstract

**Introduction:**

End-of-rotation assessments (ERAs) completed by clinical faculty supervising medical students are an important component of medical student performance during clinical rotations. The quality and quantity of the formative and/or summative comments provided by faculty to students on ERAs vary. The goal of this study was to better understand the experiences, limitations, and barriers that may affect faculty at a single institution and its affiliated sites when completing this assessment.

**Methods:**

A qualitative study design was used, with phenomenology as the qualitative design of inquiry. Clinical faculty at 3 student rotation sites who worked with students and had filled out the electronic assessment form were asked to participate. A virtual platform was used to conduct semistructured interviews. Transcripts of the recorded interviews were reviewed and analyzed to identify emerging and recurrent themes.

**Results:**

Eleven faculty members (8 men and 3 women) were interviewed. Most participants felt that the time spent with medical students was limited, compromising the assessment process—particularly at sites where they are assigned to inpatient service for 1 week at a time. Longer intervals between end-of-rotation and completing the assessment limited details in the narrative components. Some participants were hesitant to assign students lower scores and to write negative comments in their assessments. Although constructive comments could be provided verbally, they were not always stipulated as comments on the assessment form. Many were concerned that written comments could negatively affect a student's future career. The participants recognized the importance and benefit of writing comments specific to the individual student. Many opined that providing prewritten examples of suggested comments would result in a generic assessment.

**Discussion:**

The experiences, limitations, and barriers that affected faculty members' ability to assess medical students at the end of the neurology rotation included limited time spent with students, a longer time taken to fill out the assessment form, and reluctance to write negative comments that could potentially affect a student's career. Specific comments about individual students were deemed important. Shorter and more frequent assessments, modifications to faculty schedules, faculty development initiatives, and adoption of a growth mindset are potential ways to overcome barriers faced by faculty.

## Introduction

End-of-rotation assessments (ERAs) are an important depiction of a medical student's clinical performance during clinical rotations throughout medical school. These assessments are completed by clinical faculty who supervise medical students in the inpatient and outpatient settings. Clinical faculty are tasked with providing specific feedback to medical students based on their observed performance, using forms that consist of a rating scale and/or a narrative section. The quality and quantity of the formative and/or summative comments provided by clinical faculty to students vary.

Feedback has been defined as “specific information about the comparison between a trainee's observed performance and a standard, given with the intent to improve the trainee's performance.”^1,2^ The RIME (reporter, interpreter, manager, and educator) framework is a tool for narrative feedback.^3^ This tool was devised to improve written assessment by classifying trainees' behaviors and skills into 4 observed domains. Hanson et al.^4^ propose that an assessment system that uses comments based on learner observation, and the RIME framework would be more effective at assessing whether a learner is able to appropriately perform the activities required to be an effective physician in their chosen field.

In one study, the narrative-based ratings of student competence by a grading committee from an internal medicine rotation correlated with examination scores.^5^ Narrative assessments converted by a small group of experienced graders were at least as reliable as numeric scoring by evaluators in another study.^6^ In a third study, residents who required additional attention early on in residency were identified based on narrative comments from only several collected reports.^7^

Assessment forms filled out by clinical faculty at academic institutions typically consist of competency-based questions or prompts that are answered using a Likert scale and/or a narrative section. Our ERA form, entitled Student Performance Evaluation, consists of 15 items that are rated using a 5-point Likert scale. There are 2 additional comment fields where formative and summative feedback can be provided (eAppendix 1, links.lww.com/NE9/A55). Feedback that is detailed and specific makes a greater contribution to students' continued progress and development.^8,9^ After performing a content analysis on the narrative section of medical students' assessment forms following a pediatric clerkship, the comments were infrequently related to basic clinical skills and were not specific enough to lead to effective change in a student's performance.^8^

We identified a disparity in the content of the comment fields designated for formative and summative feedback. Some comments are detailed and specific, while others are brief and nonspecific. The goal of this study was to better understand the experiences, limitations, and barriers that may be affecting faculty at our institution and its affiliated sites—as they complete the student assessment form. Gaining insight into those experiences and limitations could help guide changes to the clerkship and/or revisions to the form.

## Methods

A qualitative study design was used for this study, with phenomenology as the qualitative design of inquiry.^10^ Participants were recruited using a purposive sampling technique given that the most important criterion is the participant's experience with the phenomenon under study.^10,11^ Twenty-five faculty members from 3 training sites involved with assessing medical students during the neurology rotation were invited to participate. Those invited to participate had faculty appointments in the School of Medicine and were required to have filled out the electronic ERA. Residents and those who have never filled out the electronic form were excluded. Invitations were sent by email with an attached information sheet. The email invitation was resent to faculty who did not respond to the initial email.

A virtual platform (WebEx) was used by 2 of the researchers (M.C.E. and J.M.-R.) to conduct in-depth semistructured interviews. The interview protocol was devised by all 3 of the researchers as a guideline for asking questions during the interviews (eAppendix 2, links.lww.com/NE9/A56). The questions pertained to factors that could affect the experience of filling out an ERA form for medical students, including potential barriers. One of the interviewers (M.C.E.) has been the Clerkship Director for 8 years and received formal and informal training in conducting interviews as part of their graduate training. The second interviewer (J.M.-R.), who has been the Clerkship Site Director for 5 years and an Associate Program Director for 6 months, received informal training in conducting interviews.^12,13^ The supervising author (B.A.A.W.) is a faculty member at a graduate program who has received formal training in qualitative research and has experience conducting interviews.

To ensure continuity, both the interviewers (M.C.E. and J.M.-R.) attended 8 of the 10 interview sessions. The interviewers took notes and recorded the interviews from the virtual platform. Recorded conversations and notes were the forms of data acquired from faculty members. Asking for feedback from the participants about the data or their interpretation of the data, or “member checking,” is one of the ways the authors (M.C.E. and J.M.-R.) established credibility.^14^ This was performed throughout the interview process or as a summary of comments made by the participants at the end of the session. The interviewers debriefed immediately following the interviews and discussed the themes that emerged from each session. The transcripts were revised and corrected after each interview. Recurrent themes were acknowledged by the interviewers with each subsequent interview, based on the content of the interview and review of the previous transcripts. This informal data analysis performed along with data collection helped determine when saturation was reached. Although saturation was thought to have been achieved by the 10th faculty interview, the 11th participant volunteering to take part in the study was interviewed.

A list of codes was outlined based on the evolving themes that were recognized during the informal data analysis. Once the interviews ended, personal identifiers were removed, and the authors independently reviewed the transcribed data in aggregate. The data were coded by “bracketing chunks,” which entailed writing a word representing a specific category in the margins.^10,15^ The authors met to discuss the codes, significant statements, and themes that were identified. Disagreements were reviewed with the supervising author. Sample statements related to the various themes quoted by the interviewers are outlined in the results section.

When considering reflexivity, both of the interviewers took into account the potential influence that their roles as Clerkship and Site Directors could have on the study. The interviewers were careful not to influence the participants' responses to questions during the interviews. Reflexivity was also important during data analysis so that the researchers' roles in the clerkship did not directly influence their interpretations of the themes along with the meaning ascribed to those themes. The interviewers' interpretations of the themes and their ascribed meanings were reviewed with the supervising author (B.A.A.W.) who did not have a role in the clerkship.

### Standard Protocol Approvals, Registrations, and Participant Consents

This study was approved by the institutional review board and determined to be exempt (based on exempt category 2—research involving educational tests, surveys, interviews, or observations). Participation was voluntary, and agreeing to participate in the interview implied voluntary consent. This was reiterated at the beginning of all interviews.

### Data Availability

Anonymized data not published within this article is available upon request from any qualified investigator.

## Results

Eleven faculty members—8 men and 3 women—agreed to participate in the interviews. A clerkship site director, 2 fellowship directors, and an associate program director were among those who volunteered. Eight of the faculty members were assistant professors and 3 were associate professors. Their years in practice ranged from 3 to 27.

### The Assessment Process as a Whole and the Factors Influencing It

The participants reported that their experience with the assessment process was positive, and they agreed that student assessments are valuable and necessary. Some faculty members described group dynamics and different student personalities as factors that could influence their assessment of students. Students who were more engaged and eager to answer questions were easier to assess. Other students have to be directly addressed if the evaluator wanted to assess their medical knowledge and understanding of a particular topic.

There’s sometimes also a group dynamic, whether there’s 1 student, who is very vocal, and may overshadow the other ones. Others are also very good or even better, but you can’t tell, because one may overshadow the other. Sometimes there’s 1 student that’s very strong, and the other 1 rides on that person’s coattails, and they chime in at the right time where it seems like they are interested. Also, the residents that are rotating on the team may change the dynamic.

There are different personalities, and this is a factor that influences a lot. Some of the students are very vocal and will express their opinion, even if they are not sure about something, even if they are wrong. Some of the students will stay very quiet until they are prompted to answer the questions. Oftentimes those who are very quiet, when I ask a question, they actually know [the answers to] almost everything I ask, but they are quiet. So, I would say that the person who is evaluating [students] has to be watchful about differences in their personalities (Table 1).

**Table 1:** Summary of Themes and Quotes: Assessment Process and Influencing Factors

Theme	Key quote
Group dynamics	There are different personalities, and this is a factor that influences a lot. Some of the students are very vocal and will express their opinion, even if they are not sure about something, even if they are wrong. Some of the students will stay very quiet until they are prompted to answer the questions. Oftentimes those who are very quiet, when I ask a question, they actually know [the answers to] almost everything I ask, but they are quiet. So, I would say that the person who is evaluating [students] has to be watchful about differences in their personalities.
Time limitations: Time with students	I think it is hard when we have a limited amount of time to get to know these students, and the way that our services are set up it is hard to have, I think, a real relationship with a student—it's very surface.
Time limitations: Completing the evaluation	Those questions where you click are easier to answer. It's much more difficult to invent something on your own. However, I think that comments [in the narrative component of the end-of-rotation assessment] that you generate on your own are probably more valuable in the sense that you have to think about what you [are going to] write. And you actually put something specific about a specific person that is not generic in the sense that you just click something. If there are a lot of good things to write, it's easy. It can be difficult if there are not many good things [to write].
Timing of the evaluation	I always feel that the timeline between working with the student and the evaluation should be short because I will have fresh memories of a particular student in a particular scenario. When it takes too long for me to evaluate a student, I have more trouble remembering their performance in detail.

Most of the participants felt that the time spent with the medical students was limited, which many felt compromised the assessment process. Faculty at the sites where they are assigned to inpatient service for 1 week at a time reported particular concern about not being able to spend enough time with the student during that week. The participants noted that the narratives could be more valuable and detailed if they spent more time with the students.

I think it is hard when we have a limited amount of time to get to know these students, and the way that our services are set up it is hard to have, I think, a real relationship with a student—it's very surface (Table 1).

Sometimes the student may not round long enough with us during the rotation. So, it will be difficult for me to give a really clear evaluation for them if they didn't round with us fully. Usually, if they round with us the whole week or 4 days, I will be able to tell. Sometimes they have other stuff like lectures or [other] things, and then the rotation with us will be shorter. For those [students] it can be difficult [to evaluate]. But I don't think that's a problem with the [assessment] process itself.

The participants noted that although it takes more time to write comments in the ERA, they recognize that writing specific comments about an individual student is important and beneficial to the learner.

Those questions where you click are easier to answer. It's much more difficult to invent something on your own. However, I think that comments [in the narrative component of the ERA] that you generate on your own are probably more valuable in the sense that you have to think about what you [are going to] write. And you actually put something specific about a specific person that is not generic in the sense that you just click something. If there are a lot of good things to write, it's easy. It can be difficult if there are not many good things [to write] (Table 1).

The participants shared that the longer the time interval between working with the student and completing the ERA, the more likely they would forget details about the individual student. As such, the comments that are written would be more generic and less specific. All the participants advocated for receiving the ERA as soon as possible following their interaction with a student.

I always feel that the timeline between working with the student and the evaluation should be short because I will have fresh memories of a particular student in a particular scenario. When it takes too long for me to evaluate a student, I have more trouble remembering their performance in detail (Table 1).

### Grade Inflation: Wanting to Help the Students

The participants explained that they had a natural inclination to want to help students by providing mainly positive comments. While they might provide constructive comments to students verbally, they were less inclined to write such comments in the ERA itself.

I also have in the back of my mind that for these students, particularly if they want to go into neurology, everyone's got to be above average. So that plays into how I evaluate students.

[The students] do meet expectations, but the way that grade inflation works, you can't just pass something. That's almost bad. I think a lot of us know that and find it very hard to use these evaluations constructively knowing that background (Table 2).

**Table 2:** Summary of Themes and Quotes: Grade Inflation

Theme	Key quote
Wanting to help students	[The students] do meet expectations, but the way that grade inflation works, you can't just pass something. That's almost bad. I think a lot of us know that and find it very hard to use these evaluations constructively knowing that background.
Avoidance of negative comments	It's always easier to provide positive feedback than negative feedback. When I'm providing negative feedback, it requires more energy from me because I want to be constructive. We don't want to hurt people…makes me edit the text, measure words and see how things can be phrased in a in a better way…
Weakening of the evaluation	[The assessment process] is valuable but I guess it's only as valuable as the data you can collect. So, if everyone just rates every medical student as 5 out of 5, and they did a great job, then I guess it's not so helpful. So having good data and good metrics to measure are important.

Most of the participants volunteered their reluctance to write “negative comments” in the ERA. Many were concerned these could negatively affect a student's future career. Some participants acknowledged they were worried that such comments would not be well-received by students, including the possibility of litigation, if not properly framed.

It's difficult to know what to write if there aren't good things to write.

It's always easier to provide positive feedback than negative feedback. When I'm providing negative feedback, it requires more energy from me, because I want to be constructive. We don't want to hurt people…makes me edit the text, measure words and see how things can be phrased in a in a better way… (Table 2).

We are cognizant about the effect that our evaluation can have on the future career of the medical students, so I usually avoid anything categorical or strong worded. There's also litigation potential for strongly worded statements that can affect the student's future [which] is in the back on my mind as well.

Feedback—even positively framed constructive feedback—is not always taken very well, especially when you don't know the student very well.

Although it is stipulated that the comments in the section entitled “formative feedback” would not be copied into the Medical Student Performance Evaluation and would be used for the purpose of continued student growth and improvement, faculty members still expressed reluctance to write comments that could be perceived as negative. Certain participants noted that the formative feedback section of the ERA was not the best or only way to provide constructive feedback. Some commented that one should take into consideration that feedback is also given verbally.

I think people worry about anything in box 16 (entitled “formative feedback”; eAppendix 1, links.lww.com/NE9/A55) a lot. I think all of us sort of worry about where is that going to go, even if it’s a blandly constructive thing. Where is that going? Where’s that really going to go?

“Grade inflation” leading to a student assessment that is less valuable was a concern for certain participants.

[The assessment process] is valuable but I guess it’s only as valuable as the data you can collect. So, if everyone just rates every medical student as 5 out of 5, and they did a great job, then I guess it’s not so helpful. So having good data and good metrics to measure are important (Table 2).

### The Assessment Form Itself

Faculty members found that having the student’s picture on the ERA form was helpful. The questions with Likert scoring were described as easier to answer. Definitions outlining what constitutes a particular score on the Likert scale were reported as useful. A few of the participants noted that they had difficulty delineating which student should get a score of 4 vs one who gets a score of 5 in the various competencies.

Most of the students [perform] above expectations most of the time. They are very impressive, interested, they study hard, and they are looking for learning opportunities. So, for me a score of 4 or 5 is always difficult to judge… and I have a little bit of a dichotomy. The better the definitions of what constitutes a 4 or 5, the easier it would be for me (Table 3).

**Table 3:** Summary of Themes and Quotes: The Assessment Form

Theme	Key quote
Clear definitions of the scoring system	Most of the students [perform] above expectations most of the time. They are very impressive, interested, they study hard, and they are looking for learning opportunities. So, for me a score of 4 or 5 is always difficult to judge… and I have a little bit of a dichotomy. The better the definitions of what constitutes a 4 or 5, the easier it would be for me.
Importance of narrative sections	Faculty need to feel free to provide comments whichever way they want because every student is different.
Method of assessment delivery	If it's possible to move directly from a link into the [assessment] page without a password…that will make it easier to fill it [out] directly on my phone…easier electronic access to the form [could make it easier to use].
Applicability of items on the form	There are some things that are often pretty hard to evaluate, I think—like advocates for patients…cost-effective care. That may be hard to assess during the rotation, or particularly if you just work with a student for a week or so. Maybe if there's a standout person that shared their insights on a particular treatment modality or options…it's otherwise hard to gauge really if they met expectations or if they're a little bit better than that. Some of the questions—like presentation, writing notes, professionalism, and medical knowledge, are a little bit easier to answer in the time that you have [to spend with the students].
Utility of prewritten, competency-based comments	…too cookie cutter; less personal; or too generic. Having to come up with comments for the narrative sections encourages the evaluator to think rather than clicking mindlessly.

Although the participants acknowledged that it takes more time to write comments about an individual student, they opined that having the option to write comments, is valuable. One faculty member noted the following: “Faculty need to feel free to provide comments whichever way they want because every student is different” (Table 3).

Most of the participants indicated that they would not change the form; the length of the form was appropriate with only a few stating that it was too long. While all the participants thought that an electronic form was easier to access, some cited technical difficulties including the need to use a password, password expiration, lack of familiarity with the website, and glitches on the website as barriers. One faculty member recommended using the platform used to fill out the resident assessments (Blackboard) to complete those of students.

Blackboard is easy because from the email, you just click the link. If it could be done on Blackboard, I think that would improve compliance, just because of how often Blackboard sends reminders and maybe other people's familiarity with that software.

While some faculty members noted that a paper assessment would be more convenient because it could be filled out in real time, others noted that they would like to have easier access to the form on their smart phones.

If they just have the paper, you can just check it off.

If it’s possible to move directly from a link into the [assessment] page without a password…that will make it easier to fill it [out] directly on my phone…easier electronic access to the form [could make it easier to use] (Table 3).

Most of the participants noted that certain items in the ERA were difficult for them to assess, given the length of their interaction with the students. They also noted that those assessment items did not align with student responsibilities during the neurology rotation; specifically, items relating to advocating for patients to access health care services and assistance, considering cost-effectiveness in developing diagnostic and treatment strategies, and collaborating and coordinating with different members of the health care team. While the student's interaction with the immediate neurology patient care team is observed, the student's interaction with other professional team members can be difficult for the evaluator to have specifically observed.

Sometimes when I'm the answering questions [on the ERA], a few of them can feel like they don't really fit with the experience the students have had.

There are some things that are often pretty hard to evaluate, I think—like advocates for patients…cost-effective care. That may be hard to assess during the rotation, or particularly if you just work with a student for a week or so. Maybe if there's a standout person that shared their insights on a particular treatment modality or options…it's otherwise hard to gauge really if they met expectations or if they're a little bit better than that. Some of the questions—like presentation, writing notes, professionalism, and medical knowledge are a little bit easier to answer in the time that you have [to spend with the students] (Table 3).

When the participants were asked about having the option to select from a sample of prewritten, competency-based comments, they all noted that this would make filling out the narrative component easier and would increase the total word count in the narrative components of the ERA. However, the specific comments unique to the individual student would be lost. They described that the ERA would be “too cookie cutter; less personal; or too generic.” One faculty member stated: “Having to come up with comments for the narrative sections encourages the evaluator to think rather than clicking mindlessly” (Table 3).

Several participants suggested having both options, noting that those who have a tendency to write detailed and specific comments would continue to do so. However, the faculty members who find it more difficult to write detailed comments might choose from the sample of prewritten comments, perhaps resulting in a more meaningful student assessment. Some participants noted that though they would not click on the prewritten comments, having those comments as part of the ERA would serve as a guide about the types of statements they can use to describe a student's performance. One participant specifically stated that those statements would help guide their specific comments about a student given that English is not their first language.

Another suggestion from several participants was to add “hard stops” after each competency so that the faculty members would be reminded to add specific comments pertaining to the assigned score. Some suggested that this can be done for certain competencies so as not to discourage evaluators with a longer ERA form and the need to provide comments after every competency.

I think if after every [assessment] item, there's a box underneath for attendings to give feedback again, I am not sure if the evaluators will actually do that, but at least it gives them an option to write something based on the number they chose. Though, I think people like to keep it simple—give them more boxes it's going to get more frustrating for them.

On average, the participants reported that they took 5–15 minutes to fill out the ERA. Ten of the 11 faculty participants interviewed noted that a lack of time was not a limiting factor to writing more detailed and specific comments in the narrative sections. The remaining participant noted that during instances when they are required to fill out assessments for several learners on the team, this tends to limit the amount that is written in the narrative sections of the various forms. Table 1 lists a summary of themes and quotes from this study.

## Discussion

This study aimed to better understand the experiences, limitations, and barriers that may be affecting neurology clinical faculty members at 3 different sites as they complete the student ERA including the narrative assessment. A qualitative study design was used for this study, with phenomenology as the qualitative design of inquiry.

The importance and benefits of detailed and specific narrative comments have been previously documented.^2-9,16-18^ This study adds to the understanding of the barriers of reporting such comments by using a qualitative methodology, which allowed for a deeper understanding of the experiences of clinical faculty. We found that time was a major barrier in many ways. While the time taken to fill out a student's ERA was not considered a limiting factor, many participants noted that the assessment process could be more valuable if they were able to spend more time with the individual students. The participants also noted that a shorter time interval between working with the student and filling out the ERA allowed them to write more specific comments. Shorter assessments, done more frequently, at the end of every week might be beneficial in addressing this concern. Weekly feedback forms that address the student's performance with specific examples could provide more continuity. The attending physician filling out the student's ERA could use those weekly assessments when completing the ERA including the narrative comments to depict the student's progress throughout the rotation. The importance of continuity can also be taken into consideration when faculty schedules are devised.

The participants commented about the tendency to ascribe higher scores and to write positive comments. Some participants noted that while they would provide constructive feedback to medical students verbally, they were less inclined to write such feedback on the assessment form. Several of the participants reported concern about potential litigation associated with perceived negative comments. None of the participants offered possible solutions to this conundrum. Multipronged faculty development initiatives might provide faculty with the appropriate tools and confidence required to offer both positive and constructive feedback.^19^ An assessment form that requires constructive comments (some of which could be provided) is another potential solution. A specific observation that supports the constructive comment could also be requested. Changes that promote Dweck's self-theory and a growth mindset in learners, faculty, and our medical educational systems is another important consideration. An educational alliance that adopts a growth mindset would allow faculty to provide constructive feedback to learners in favor of continued growth and improvement as the learner advances toward mastering various competencies. Contrarily, a fixed mindset placing emphasis on scores and performance orientation results in a culture where learners and faculty feel compelled to conceal areas requiring further development.^20^

All the participants noted that although having samples of prewritten comments for the narrative component of the ERA would make it easier for faculty to fill out this part of the form, those comments would not be unique to the individual students. Three faculty members noted that they would appreciate this option as a guide to writing comments in the narrative sections, particularly when English is not their first language.

Two particular items on the ERA form were cited by many of the participants as difficult to assess during the neurology rotation. Revisions to the form so that the components are clearly aligned with observable student performance may be considered.

Some faculty members suggested increasing the number of required comment fields in the form. Those same participants cautioned that this modification could make the ERA more onerous leading to vaguer comments or faculty declining to complete the form. One study assessed the effect of increasing total comment fields, finding that the proportion of constructive comments were lower with the revised form.^16^

A qualitative study exploring the challenges faced by faculty in providing trainees with meaningful assessment and feedback in a Canadian surgical residency training program was identified during a literature search.^17^ Some similar themes were described including faculty being reluctant to provide constructive feedback or report poor performance, increased effort in completing assessments for poorly performing learners, insufficient interaction with learners to complete an assessment, and fear of legal action due to negative comments.

Some themes reported in the study by McQueen et al.^17^ were not identified in this study. The participants in this study denied that the time spent on clinical duties limited their ability to fill out the ERA including the ability to provide detailed comments. The surgeons in the study by McQueen et al.^17^ also reported a fear of being labeled as intimidating or harassing, which was not stipulated as a concern by the neurologists in this study.

There are certain limitations to our study. The purposive sampling technique inherent to this qualitative study limits the interviews to select faculty members. Transferability, or the extent to which the findings of this study can be applied to different settings, was limited in that this study was performed in the specialty of neurology, at 3 affiliate sites, from 1 institution. Although the outcomes may not be generalizable to other specialties, the authors anticipate that the information from this study may be beneficial to clerkship directors and other medical educators. The specific form used by various medical schools varies, which could also limit the transferability of some of the findings of this study.

Conducting similar qualitative studies in other medical specialties could provide additional insight into the factors and potential limitations that faculty experience when completing an ERA for medical students. Studies to assess whether changes to the assessment form, the student clerks' schedules, or faculty schedules have a significant impact on student learning would also be insightful.

## Appendix Authors

**Table:**

Name	Location	Contribution
Marie Carl Eugene, DO, MSHPE	Neurology Department, UConn Health/UConn School of Medicine, Farmington, CT	Drafting/revision of the article for content, including medical writing for content; major role in the acquisition of data; study concept or design; and analysis or interpretation of data
Jose Montes-Rivera, MD	Neurology Department, UConn Health/UConn School of Medicine, Farmington, CT	Drafting/revision of the article for content, including medical writing for content; major role in the acquisition of data; study concept or design; and analysis or interpretation of data
Bobbie Ann Adair White, EdD, MA	MGH Institute of Health Professions, Boston, MA	Drafting/revision of the article for content, including medical writing for content; study concept or design; and analysis or interpretation of data

## Glossary

ERA: end-of-rotation assessment
RIME: reporter, interpreter, manager, and educator
